# First report of *Acinetobacter pittii* acute community-acquired pneumonia in an immunocompetent patient in France following a heat wave

**DOI:** 10.1186/s12879-023-08945-y

**Published:** 2024-01-02

**Authors:** Bérénice Souhail, Maxime Danjean, Mélanie Mercier-Darty, Giuliana Amaddeo, Anna Sessa, Vincent Fihman, Adrien Galy, Paul Louis Woerther, Raphaël Lepeule

**Affiliations:** 1 Antimicrobial Stewardship Team, Department of Prevention, Diagnosis, and Treatment of Infections, Henri Mondor University Hospital, AP-HP, 1, rue Gustave Eiffel, 94000 Créteil, France; 2 Bacteriology and Infection Control Unit, Department of Prevention, Diagnosis, and Treatment of Infections, Henri Mondor University Hospital, AP-HP, Créteil, France; 3 EnvA, DYNAMYC, UPEC, Paris-Est Créteil University, EA 7380 Créteil, France; 4 Next Generation Sequencing Platform, Henri Mondor University Hospital, AP-HP, Créteil, France; 5 Hepatology Department, Henri Mondor University Hospital, AP-HP, Créteil, France

**Keywords:** Community-acquired pneumonia, *Acinetobacter pittii*, *Acinetobacter calcoaceticus-baumannii* complex, Global warming, Emerging infectious diseases

## Abstract

**Background:**

In recent years, *Acinetobacter baumannii-calcoaceticus* complex (ABC) infections have attracted attention, mainly because of the impact of carbapenem-resistant isolates in hospital-acquired infections. However, acute community-acquired ABC infections are not uncommon in warm and humid countries, where they are responsible for community-acquired infections with specific clinical features. To date, such infection has not been reported in France.

**Case presentation:**

We report the case of a 55-year-old non-immunocompromised patient living in France with no known risk factors for community-acquired ABC infections who presented pneumonia with bloodstream infection due to wild-type *A. pittii*. The outcome was favorable after 7 days of antibiotic treatment with cefepime. We confirmed bacterial identification with whole-genome sequencing, and we examined the *A. pitii* core-genome phylogeny for genomic clusters.

**Conclusions:**

This situation is uncommon in Europe and occurred after a heat wave in France with temperatures above 38 °C. Herein, we discuss the possibility that this pneumonia may be emerging in the current context of global warming.

## Introduction

*Acinetobacter* spp. is a nonfermenting gram-negative coccobacillus. The genus is composed of over thirty species, some of which are genomically close and clustered into complexes, such as the *Acinetobacter baumannii-calcoaceticus complex* (ABC), which is composed of *A. baumannii, A. nosocomialis, A. calcoaceticus and A. pittii* [1]. In the clinical laboratory, ABC cultivation is easy since it grows overnight on standard media under ambient atmosphere. Even though its reservoir is environmental, this bacterium is involved in healthcare-associated diseases in hospitals [2] because of factors enhancing host colonization and infection [3]. In addition, ABC shows increasing rates of antimicrobial resistance, setting this genus as one the ESKAPE list of priority pathogens with multidrug resistance and high virulence for which new antibiotics are urgently needed [4].

In Europe, ABC is typically involved in nosocomial infections in intensive care units, such as ventilated acquired pneumoniae and catheter-related infections [5, 6]. In contrast, it is an emerging cause of community-acquired pneumonia, especially during the warm and humid months in some tropical and subtropical countries of Asia and Oceania [7, 8].

Community-acquired infections with ABC are characterized by clinical features distinctive from nosocomial infections [9]. Indeed, whereas nosocomial pneumonia generally has an insidious onset, community-acquired pneumonia caused by ABC is usually characterized by a rapid and fulminant onset associated with sepsis, organ failure and a high mortality rate [7, 8].

## Case presentation

Here, we present the case of a 55-year-old non-immunocompromised male patient born in Vietnam and living in France who had no history of travel abroad since 2010. His main past medical history included type 2 diabetes, treated with metformin (HbA1c < 7%), well-controlled high blood pressure with angiotensin-converting enzyme inhibitor (perindopril) and calcium channel blockers (amlodipine) and dyslipidemia treated with atorvastatin. He had no history of surgery, smoking, alcohol or drug abuse. He was retired in 2017 (former archivist), had no children and lived in the Paris area in an apartment with his wife. He had no pets at home.

During his vacation in the New Aquitaine region (southwest side of France), he developed fever (up to 39 °C) with chills. The patient had no other symptoms throughout 4 days. Once back home, he consulted his general practitioner for persistent fever. The biological analysis prescribed showed biological inflammation with CRP = 121 mg/L and leukocytes = 19 G/L with polynuclear neutrophil predominance, acute kidney failure (creatinine = 255 µmol/L) and cytolysis up to 5 times normal (ASAT = 178 UI/L, ALAT = 250 UI/L) with anicteric cholestasis (PAL = 216 UI/L and GGT = 190 UI/L). The patient was then referred to the emergency department of our hospital. The clinical examination was normal apart from the persistence of fever, he had no signs of severity and the qSOFA score was 0. An abdominal ultrasound was performed, which showed no renal abnormalities and no dilation of the intra- or extrahepatic bile ducts. Two blood cultures were drawn, and the patient was admitted to the hepatology department, where no empiric antibiotic therapy was initiated in the absence of signs of severity.

The evolution was marked by the persistence of an isolated fever with chills. At the same time, renal function improved with rehydration, and the liver function test improved spontaneously. Blood cultures taken at entry and on subsequent days were positive for wild-type *Acinetobacter pittii* (3 blood culture samples taken 24 h apart). Standard MALDI-TOF (Bruker®) identification from bacterial colonies on solid media culture was confirmed with *rrs* gene sequencing (Molzym®, Germany) and identified *A. pittii*, a species belonging to the *A. baumannii-calcoaceticus* complex. The strain displayed a wild-type antibiotic resistance phenotype according to the disk-diffusion method on Mueller-Hinton agar, as recommended by the French Committee for Antimicrobial Susceptibility Testing (Table 1).

**Table 1:** Antibiotic susceptibility of the *A. pitii* strain isolated

Amoxicillin	R
Amoxicilline – clavulanate acid	R
Ticarcillin	S
Ticarcillin – clavulanate acid	S
Imipenem	S
Meropenem	S
Ertapenem	R
Cefalexin	R
Cefotaxime	R
Cefepime	S
Tobramycin	S
Amikacin	S
Gentamicin	S
Fosfomycin	R
Trimethoprim/Sulfamethoxazole	S
Levofloxacin	S
Ciprofloxacin	S (increased exposure)

HIV, HAV, HBV, HCV and HEV serologies were negative. EBV and CMV serologies showed a pattern of previous infection. SARS CoV-2 PCR on nasopharyngeal swab was negative.

**Fig. 1:**
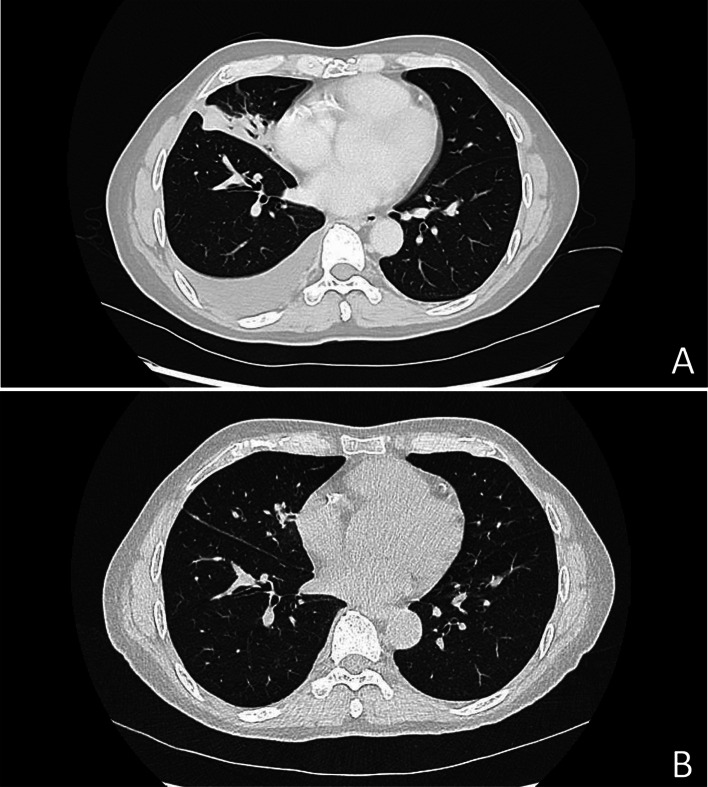
(**A**) Chest CT scan on day 5 of admission showing alveolar consolidation with air bronchograms in the middle lobe and right pleural effusion of moderate size. (**B**) Chest CT scan at the 3-month follow-up showing partial regression of alveolar consolidation and complete regression of the right pleural effusion

Antibiotic therapy with cefotaxime was started when the blood culture was positive for gram-negative bacilli (time to positivity 11, 9 h in aerobic environment) and then changed to cefepime 2 g/8 h after bacteriological identification. The exploration of the portal of entry and staging for extension included a thoracic-abdominal-pelvic CT scan on day 5, which showed alveolar consolidation with air bronchograms in the middle lobe associated with a right pleural effusion of moderate size (Fig. 1A). No other abnormalities were detected. Because the patient had already received 5 days of antibiotic treatment and did not show any pulmonary symptoms, no sputum culture was performed. Transthoracic echocardiography was performed on day 7 and did not show endocarditis signs. The evolution was rapidly favorable with antibiotic treatment: the patient became afebrile within 48 h, the biological inflammatory syndrome decreased, and the control blood cultures were negative. Antibiotic treatment with cefepime was continued for 7 days. The patient was discharged home at the end of the antibiotic treatment. At 3 months, the evolution of the pulmonary infection was favorable at the clinical, biological and radiological levels (Fig. 1B).

**Fig. 2:**
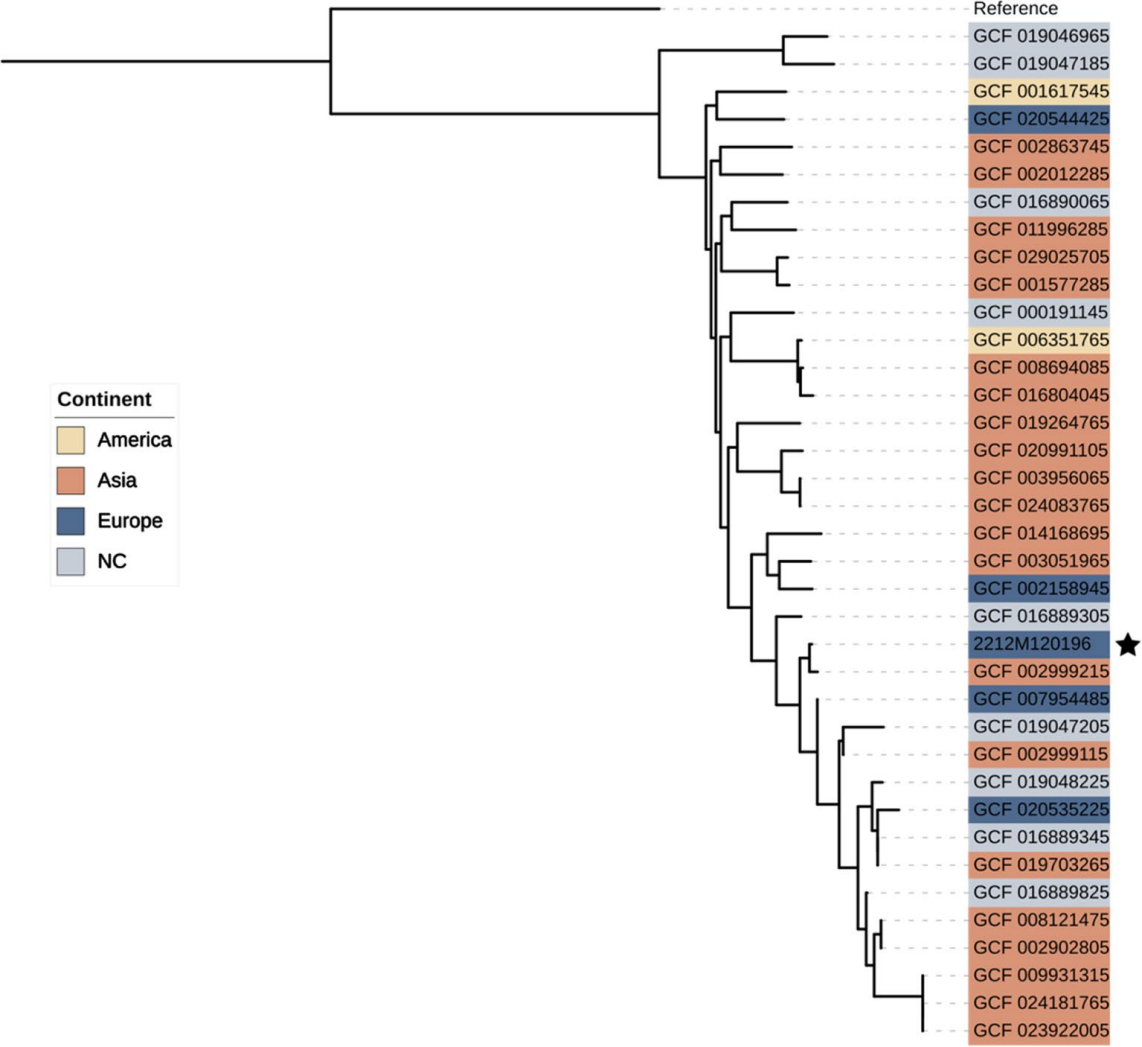
Core-genome phylogeny of *A. pitti* isolates. Phylogenetic tree of 38 *A. pittii* isolates combining 36 previously published genomes (assembly level: chromosome and complete genome), 1 reference (*A. baumannii* – GCF_008632635*)* and the current clinical isolate (2212M120196), which is marked with a black star. The latter does not cluster independently of the others, suggesting that it is likely to be only slightly distinct from the others in terms of its core genome. Furthermore, metadata providing information on the country of isolation of the strain did not establish a link between geographic origin and the associated genetic background. NC: unknown country of isolation

To further understand this unusual infection, we confirmed bacterial identification with whole-genome sequencing (Illumina®). Pair-end reads were *de novo* assembled with the SPAdes-based [10] Shovill algorithm (https://github.com/tseemann/shovill). Species identification was performed using the KmerFinder approach [11] in combination with phylogeny within the bacterial species belonging to ABC. We identified two beta-lactamase-encoding chromosomal genes, *bla*_ADC− 25_ and *bla*_OXA− 500,_ by querying the Resfinder database [12], which is consistent with phenotypic data. We computed the core genome using Roary [13] using a MAFFT alignment. Maximum-likelihood phylogenies using Generalize Time Reversible model were set with PhyML [14] into (i) ABC isolates (data not shown) and then (ii) *A. pittii* isolates (Fig. 2), including external genomes previously published.

## Discussion and conclusions

This case is of particular interest because of the clinical presentation, which is unusual regarding the classically described cases of community-acquired infections with ABC in tropical countries [9, 15]. Despite blood stream infection and delayed appropriate antibiotic treatment, the patient did not experience fulminant infection, organ failure or septic shock.

To our knowledge, this is the first case report of a community-acquired infection with *A. pittii* in an immunocompetent patient in France. In 2017, a French group reported the case of a 45-year-old woman with a history of smoking and systemic lupus, with community-acquired cavitary pneumonia due to *A. pittii* [16]. In our case, apart from well-treated diabetes mellitus, our patient had no known risk factors for community-acquired ABC infection, such as excessive alcohol consumption, smoking, chronic lung disease or living in a tropical or subtropical climate [17].

Data from the literature strongly hypothesized that ABC inhabits a warm and humid environment, as suggested by several studies conducted in Taiwan, Singapore and Australia, which reported that more than two out of three cases of community-acquired ABC infections occurred during the hot and humid months [8, 15, 18]. Interestingly, this case occurred just after a remarkably hot summer period in France, in a wet area (Gironde estuary, close to the ocean), with unusually high temperatures for the region and the time of the year (above 38 °C with a peak at 43 °C) [19].

Data from our *A. pittii* isolate core-genome phylogeny showed no predominant clusters or relationship between the different perceived clades and the country of origin. Thus, these data seem to point to exogenous risk factors that should be confirmed by additional observations.

Whether our case could be a consequence of global warming remains hypothetical. This observation underlines the importance of being attentive to the evolution of local epidemiology while acknowledging the variations linked to climate change. We may need to modify our empiric antibiotic therapy to take into account these emerging species if this situation becomes widespread. On a larger scale, global climate change leads to a modified epidemiology of pathogens in a multifactorial pattern [20, 21]: at the level of reservoir ecology but also in vector-borne pathologies. In the field of infectious diseases, some reports have pointed out the influence of global warning in the increasing emergence of unusual pathogens such as *Vibrio parahaemolyticus* [22, 23] and *Borrelia burgdorferi* [24] in northern countries. These reports probably reflect a new era in the field of infectious disease: the emergence of new or rare pathogens. From this perspective, the link with climate change forces us to rethink the understanding of infections and their distribution in time and space.

## Data Availability

The data used and analysed during the current study are available from the corresponding author upon reasonable request.

## References

[1] Almeida LA, Araujo R (2013). Highlights on molecular identification of closely related species. Infect Genet Evol.

[2] Eveillard M, Kempf M, Belmonte O, Pailhoriès H, Joly-Guillou ML (2013). Reservoirs of *Acinetobacter baumannii* outside the hospital and potential involvement in emerging human community-acquired Infections. Int J Infect Dis.

[3] Ibrahim S, Al-Saryi N, Al-Kadmy IMS, Aziz SN (2021). Multidrug-resistant *Acinetobacter baumannii* as an emerging concern in hospitals. Mol Biol Rep.

[4] Tacconelli E, Carrara E, Savoldi A, Harbarth S, Mendelson M, Monnet DL (2018). Discovery, research, and development of new antibiotics: the WHO priority list of antibiotic-resistant bacteria and Tuberculosis. Lancet Infect Dis.

[5] Chusri S, Chongsuvivatwong V, Rivera JI, Silpapojakul K, Singkhamanan K, McNeil E (2014). Clinical outcomes of hospital-acquired Infection with Acinetobacter nosocomialis and Acinetobacter pittii. Antimicrob Agents Chemother.

[6] Gaynes R, Edwards JR, National Nosocomial Infections Surveillance System (2005). Overview of nosocomial Infections caused by gram-negative bacilli. Clin Infect Dis.

[7] Asai N, Sakanashi D, Suematsu H, Kato H, Watanabe H, Shiota A (2019). Clinical manifestations and risk factors of community-onset Acinetobacter species Pneumonia in Japan; case control study in a single institute in Japan. J Infect Chemother.

[8] Chen MZ, Hsueh PR, Lee LN, Yu CJ, Yang PC, Luh KT (2001). Severe community-acquired Pneumonia due to *Acinetobacter baumannii*. Chest.

[9] Leung WS, Chu CM, Tsang KY, Lo FH, Lo KF, Ho PL (2006). Fulminant community-acquired *Acinetobacter baumannii* Pneumonia as a distinct clinical syndrome. Chest.

[10] Prjibelski A, Antipov D, Meleshko D, Lapidus A, Korobeynikov A (2020). Using SPAdes De Novo Assembler. Curr Protoc Bioinformatics.

[11] Clausen PTLC, Aarestrup FM, Lund O (2018). Rapid and precise alignment of raw reads against redundant databases with KMA. BMC Bioinformatics.

[12] Bortolaia V, Kaas RS, Ruppe E, Roberts MC, Schwarz S, Cattoir V (2020). ResFinder 4.0 for predictions of phenotypes from genotypes. J Antimicrob Chemother.

[13] Page AJ, Cummins CA, Hunt M, Wong VK, Reuter S, Holden MTG (2015). Roary: rapid large-scale prokaryote pan genome analysis. Bioinformatics.

[14] Guindon S, Dufayard JF, Lefort V, Anisimova M, Hordijk W, Gascuel O (2010). New algorithms and methods to estimate maximum-likelihood phylogenies: assessing the performance of PhyML 3.0. Syst Biol.

[15] Ong CWM, Lye DCB, Khoo KL, Chua GSW, Yeoh SF, Leo YS (2009). Severe community-acquired *Acinetobacter baumannii* Pneumonia: an emerging highly lethal Infectious Disease in the Asia–Pacific. Respirology.

[16] Larcher R, Pantel A, Arnaud E, Sotto A, Lavigne JP (2017). First report of cavitary Pneumonia due to community-acquired Acinetobacter pittii, study of virulence and overview of pathogenesis and treatment. BMC Infect Dis.

[17] Falagas ME, Karveli EA, Kelesidis I, Kelesidis T (2007). Community-acquired Acinetobacter Infections. Eur J Clin Microbiol Infect Dis.

[18] Davis JS, McMillan M, Swaminathan A, Kelly JA, Piera KE, Baird RW (2014). A 16-year prospective study of community-onset bacteremic Acinetobacter Pneumonia: low mortality with appropriate initial empirical antibiotic protocols. Chest.

[19] En France, des vagues de chaleur plus intenses et fréquentes [Internet]. 2022 [cited 2023 Jun 4]. Available from: https://www.linfodurable.fr/en-france-des-vagues-de-chaleur-plus-intenses-et-frequentes-35414.

[20] Baker RE, Mahmud AS, Miller IF, Rajeev M, Rasambainarivo F, Rice BL (2022). Infectious Disease in an era of global change. Nat Rev Microbiol.

[21] Mahmud AS, Martinez PP, He J, Baker RE (2020). The impact of Climate Change on Vaccine-Preventable Diseases: insights from current research and new directions. Curr Environ Health Rep.

[22] Martinez-Urtaza J, van Aerle R, Abanto M, Haendiges J, Myers RA, Trinanes J (2017). Genomic variation and evolution of Vibrio parahaemolyticus ST36 over the course of a Transcontinental Epidemic Expansion. mBio.

[23] Abanto M, Gavilan RG, Baker-Austin C, Gonzalez-Escalona N, Martinez-Urtaza J (2020). Global expansion of Pacific Northwest Vibrio parahaemolyticus sequence type 36. Emerg Infect Dis.

[24] Couper LI, MacDonald AJ, Mordecai EA (2021). Impact of prior and projected climate change on US Lyme Disease incidence. Glob Chang Biol.

